# Unilateral Renal Artery Stenosis Associated with Severe Hypertension: Atypical Presentation of Tuberculosis

**DOI:** 10.4314/ejhs.v31i4.28

**Published:** 2021-07

**Authors:** Netsanet Workneh Gidi, Workineh Tesfaye, Mohammed Beshir

**Affiliations:** 1 Department of Pediatrics and Child Health, Jimma University, Jimma, Ethiopia

**Keywords:** Tuberculosis, renal artery stenosis, hypertension in children

## Abstract

**Background:**

Renal artery stenosis is a common cause of hypertension in children; however, infectious causes of renal artery stenosis are rare. Diagnosis of TB in children is challenging, causing delay in diagnosis.

**Case Presentation:**

We report a case of a 4-year-old girl who was presented with loss of consciousness and seizure of one day duration. The patient had severe acute malnutrition, symptom complex of TB and contact with adults having smear positive tuberculosis. Subsequently, her blood pressure was 200/140 mmhg. The patient was diagnosed with disseminated TB with left renal artery stenosis, severe hypertension, cardiomegaly, upper gastrointestinal bleeding, stroke and severe malnutrition. Diagnosis was confirmed with head CT scan, MRI and ultrasound of the kidneys. The patient was on antituberculosis drugs, prednisolone, nifedipine, furosemide, antiepileptic drugs and nutritional therapy. After treatment with antituberculosis drugs and other supportive care all the symptoms subsided, while the hypertension was not controlled, necessitating surgical intervention for the left renal stenosis.

**Conclusion:**

Atypical presentations of TB have to be considered especially when there is a strong contact history. Tuberculosis preventive therapy may have prevented all the complications, had it been given the moment the index cases were diagnosed.

## Introduction

Tuberculosis (TB) is among the leading causes of death from infectious disease worldwide (1). Renovascular hypertension (RVH) is hypertension resulting from a lesion that impairs blood flow to a part or all of one or both kidneys (2). Children with RVH often present with a very high blood pressure. However, some patients may have nonspecific symptoms dominated by systemic manifestation of underlying disease (3). Presentation of TB with renal artery stenosis (RAS) and associated other cardiovascular diseases in children is very rare.

**Case description**: A 4 years old female child was presented with loss of consciousness and one episode of generalized tonic colonic seizure of 24 hours duration. She had cough, fever, loss of appetite and weight loss for one month prior to the current compliant. She also had contact with known TB patients, two of her older siblings were diagnosed with smear positive TB and were treated; though the current patient was not screened for active TB nor received tuberculosis preventive therapy. She was apparently healthy, her growth and development was normal before the current illness.

At presentation she was comatose with Glasgow coma scale of 7 out of 15. Her pulse rate was 138 per minute, respiratory rate was 28 per minute, axillary temperature was 36.1 °C, but blood pressure was not measured. Her weight for height was below -3 SD, the patient did not have respiratory distress, and chest was clear on auscultation and meningeal signs were negative; there were no abnormal physical findings in other systems.

**Diagnostics**: She was diagnosed with disseminated TB involving lung & meninges clinically plus severe acute malnutrition, bacterial meningitis was also considered. Gene expert was negative from sample taken with gastric lavage twice, CBC, ESR, cerebrospinal fluid analysis, and serum electrolytes were normal.

**Therapy**: She was put on ceftriaxone, gentamycin, antituberculosis drugs, dexamethasone, phenytoin and pyridoxine and nutritional therapy.

**Follow-up and outcomes**: On the 9th day of admission she developed left side body weakness. CT scan of the brain showed parietal cortex sub-acute infarction (right parietal cortex hypo attenuated lesion seen with lose of gray white matter differentiation). In addition, she was treated for antituberculosis drug induced fulminant hepatitis and then referred to another hospital where modifying antituberculosis regimens were possible, since the patient was too sick to interrupt the drugs for longer period.

Subsequently, MRI and EEG revealed bilateral segmental territorial anterior and posterior cerebral artery infarct, and abnormal wake EEG recording. For diagnosis of ischemic stroke and seizure disorder she was put on carbamazepine. Despite the treatments she had frequent episodes of seizure and symptom complex of TB; and anti TB medications could have been resumed, but she was sent home with the carbamazepine.

After 2 months, the patient was readmitted to hospital, she was diagnosed with severe acute malnutrition, hemiparesis 2nd to ischemic stroke, cortical blindness, stage II HTN. Her blood pressure was 200/140 mmHg, chest CT showed cardiomegaly, renal function test and urine analysis was normal. She was put on furosemide, nifedipine and carbamazepine was changed to phenytoin. Afterwards, the seizure was controlled; the body weakness and her vision were improving. But the blood pressure remained high, the lowest blood pressure was 140/100 mmHg.

Subsequently, she developed upper gastrointestinal bleeding of unidentified cause, congestive heart failure and chest x-ray showed consolidation on the right lower lung field. Renovascular hypertension was considered for the severe hypertension not responding to antihypertensive drugs. On repeat ultrasound of the abdomen, high resistance flow in the ostium of the left renal artery with PSV of 129.70cm/s, left renal arterial resistance index (RI) of 0.78 and parvus-tradus waveform in the intrarenal branches indicated left renal arterial stenosis (RAS).

The patient continued antihypertensive medications, was managed for heart failure and antituberculosis drugs were restarted with prednisolone. Subsequently, the fever subsided, nutritional status improved; respiratory distress and symptoms of heart failure resolved but the patient continued to have hypertension for which renal angioplasty was indicated, which was not available in Ethiopia. Surgical removal of the affected kidney was suggested as a second option; parents declined to the surgical treatment and decided to see her with medications with close follow up and to try possible renal angioplasty abroad.

## Discussion

Aortoarteritis and subsequent stenosis have been reported with a variety of infectious agents including spirochetes, bacteria and mycobacteria (2,3) The proposed mechanism how TB leads to hypertension is said to be through triggering of immunological responses that impairs endothelial function and lead to an increased risk of cardiovascular diseases and possibly hypertension (4). The diagnosis of RAS in our case may have been detected late, as Doppler studies can detect stenotic lesions only when they are severe. Renal arteriography remains the gold standard, but is an invasive procedure and should be undertaken when the index of suspicion for RAS is high. Magnetic resonance angiography (MRA) is a time efficient and safe test when compared with conventional arteriography (2).

Children with renovascular disease are likely to have abnormalities of other blood vessels such as aorta, cerebral, intestinal, or iliac (2, 5). Our patient had several manifestations in addition to the hypertension and RAS, the stroke, upper gastrointestinal bleeding, cardiomegaly and the cortical blindness could be associated conditions, though we did not confirm the involvement of other arteries with investigation. Surgical intervention could be delayed with medical therapy, so that the surgery will be done when the child grows up fully. Favorable long-term outcomes can be achieved when intervention is managed by a team of pediatric nephrologists, interventional radiologists, and vascular surgeons (2).

Children with TB may have atypical presentation; high index of suspicion and screening is needed to make a diagnosis of TB and related complications. Measurement of blood pressure should not be missed during a routine physical examination, so that renovascular hypertension could be diagnosed without dely. All the complications could have been prevented by tuberculosis preventive therapy, had it been considered when her siblings were diagnosed with tuberculosis. Implementation of guidelines on prevention and control of TB needs evaluation and follow up.
